# Insights into the genetic architecture of haematological traits from deep phenotyping and whole-genome sequencing for two Mediterranean isolated populations

**DOI:** 10.1038/s41598-021-04436-9

**Published:** 2022-01-21

**Authors:** Karoline Kuchenbaecker, Arthur Gilly, Daniel Suveges, Lorraine Southam, Olga Giannakopoulou, Britt Kilian, Emmanouil Tsafantakis, Maria Karaleftheri, Aliki-Eleni Farmaki, Deepti Gurdasani, Kousik Kundu, Manjinder S. Sandhu, John Danesh, Adam Butterworth, Inês Barroso, George Dedoussis, Eleftheria Zeggini

**Affiliations:** 1 Division of Psychiatry, University College of London, London, W1T 7NF UK; 2 UCL Genetics Institute, University College London, London, WC1E 6BT UK; 3 Department of Human Genetics, Wellcome Sanger Institute, Hinxton, CB10 1SA UK; 4 Institute of Translational Genomics, Helmholtz Zentrum München, German Research Center for Environmental Health, Neuherberg, Germany; 5 Wellcome Trust Centre for Human Genetics, University of Oxford, Oxford, OX3 7BN UK; 6 The Primary Care Unit, Institute of Public Health, University of Cambridge, Cambridge Biomedical Campus, Box 113, Cambridge, CB2 0SR UK; 7 Anogia Medical Centre, 740 51 Anogia, Greece; 8 Echinos Medical Centre, 67300 Echinos, Xanthi, Greece; 9 Department of Nutrition and Dietetics, School of Health Science and Education, Harokopio University of Athens, Athens, Greece; 10 MRC Unit for Lifelong Health and Ageing, Institute of Cardiovascular Science, University College London, London, WC1E 7HB UK; 11 Department of Haematology, Cambridge Biomedical Campus, University of Cambridge, Long Road, Cambridge, CB2 0PT UK; 12 Department of Medicine, University of Cambridge, Cambridge, CB2 0QQ UK; 13 The National Institute for Health Research Blood and Transplant Unit (NIHR BTRU) in Donor Health and Genomics at the University of Cambridge, Strangeways Research Laboratory, Wort’s Causeway, University of Cambridge, Cambridge, CB1 8RN UK; 14 MRC/BHF Cardiovascular Epidemiology Unit, Department of Public Health and Primary Care, Wort’s Causeway, Strangeways Research Laboratory, University of Cambridge, Cambridge, CB1 8RN UK; 15 Division of Cardiovascular Medicine, British Heart Foundation Centre of Excellence, Addenbrooke’s Hospital, Hills Road, Cambridge, CB2 0QQ UK; 16 Exeter Centre of Excellence for Diabetes Research (EXCEED), Genetics of Complex Traits, University of Exeter Medical School, University of Exeter, Exeter, UK

**Keywords:** DNA sequencing, Next-generation sequencing, Genetic association study, Medical genomics, Quantitative trait, Biomarkers

## Abstract

Haematological traits are linked to cardiovascular, metabolic, infectious and immune disorders, as well as cancer. Here, we examine the role of genetic variation in shaping haematological traits in two isolated Mediterranean populations. Using whole-genome sequencing data at 22× depth for 1457 individuals from Crete (MANOLIS) and 1617 from the Pomak villages in Greece, we carry out a genome-wide association scan for haematological traits using linear mixed models. We discover novel associations (*p* < 5 × 10^–9^) of five rare non-coding variants with alleles conferring effects of 1.44–2.63 units of standard deviation on red and white blood cell count, platelet and red cell distribution width. Moreover, 10.0% of individuals in the Pomak population and 6.8% in MANOLIS carry a pathogenic mutation in the Haemoglobin Subunit Beta (HBB) gene. The mutational spectrum is highly diverse (10 different mutations). The most frequent mutation in MANOLIS is the common Mediterranean variant IVS-I-110 (G>A) (rs35004220). In the Pomak population, c.364C>A (“HbO-Arab”, rs33946267) is most frequent (4.4% allele frequency). We demonstrate effects on haematological and other traits, including bilirubin, cholesterol, and, in MANOLIS, height and gestation age. We find less severe effects on red blood cell traits for HbS, HbO, and IVS-I-6 (T>C) compared to other b+ mutations. Overall, we uncover allelic diversity of *HBB* in Greek isolated populations and find an important role for additional rare variants outside of *HBB*.

## Introduction

The number, size and shape of blood cells vary widely between individuals. The genetic architecture of these haematological traits is complex^1^. Genome-wide association studies (GWAS) have identified more than 100 common variants with relatively small effects^1–7^. Moreover, autosomal recessive mutations in the Haemoglobin Subunit Alpha and Beta genes can cause haemoglobin disorders^8^. These mutations lead to either reduced levels or abnormal structure of the haemoglobin subunits. The most common disorders are sickle cell disease, alpha and beta thalassemia^9^. Inherited anaemia is particularly prevalent in parts of Africa, the Mediterranean region, the Middle East, the Indian subcontinent, and Southeast Asia^9^. The geographic distribution overlaps regions with a history of long-standing endemic malaria^10^ and there is strong evidence that heterozygous carrier status provides a protective effect against malaria^9^.

Haemoglobin disorders represent the most common type of monogenic disorder globally^11^. With almost 500 thalassemia mutations in the HbVar database^12^ and substantial heterogeneity in disease manifestations^13^, the genetic architecture of thalassemia in particular is complex. Knowledge of the full mutational spectrum and carrier frequencies is still limited for many populations^9^. Heterozygous mutations can lead to thalassemia trait, a milder form of haemoglobin-related abnormalities. A comprehensive assessment of the physiological effects in a large sample of heterozygotes and comparisons of individual mutations is lacking for most variants. Little is known about the role of genetic variation outside of the haemoglobin genes in areas with high prevalence of haemoglobin disorders as the vast majority of previous studies has assessed common variation only and used data from individuals of European ancestries outside historic malaria regions^1–7^. Studying isolated populations offers power gains in detecting associations involving rare and low-frequency variants^14^.

We carried out whole-genome sequencing in 3,074 individuals from two Mediterranean populations, the Hellenic Isolated Cohorts (HELIC). The HELIC cohorts (https://www.helmholtz-muenchen.de/index.php?id=53481)^15–17^ consist of MANOLIS which includes individuals from the mountainous Mylopotamos villages on Crete. The Pomak cohort includes individuals who were recruited at the Pomak villages, a set of mountainous villages in the North of Greece. Genetic isolatedness has been demonstrated for both cohorts^16^. We assess the role of common and rare variation across the genome for haematological traits. We characterise the mutational spectrum for haemoglobin genes and determine the impact of mutations across the phenome in non-clinically ascertained samples.

## Results

### Novel genetic associations with haematological traits

Following quality control, data were available for 1,457 individuals for MANOLIS and 1,617 individuals for the Pomak population. Five rare, previously unreported loci were associated with haematological traits in either cohort after adjusting for multiple testing (*p* < 5 × 10^–9^) (Table 1).

**Table 1:** Association results of the lead SNPs of novel genome-wide significant loci with haematological traits.

Trait	Chr	Gene	Rs-id	Position	Type	A1*	A0*	AF*	AF Topmed	Beta	SE	*P* value
**Pomak**												
White blood cell count	2	*LOC105374834*	rs551751343	81,484,692	Intron	T	C	0.004	0.0001	1.72	0.29	4.1 × 10^–9^
	2		–	81,745,516	–	A	G	0.004	–	1.72	0.29	4.1 × 10^–9^
	2	*LOC102724542*	rs1041582657	81,759,339	Intron	A	G	0.004	0.00001	1.72	0.29	4.1 × 10^–9^
	2		rs556280089	81,954,850	Intergenic	T	C	0.004	0.00001	1.72	0.29	4.1 × 10^–9^
	2		rs989421555	82,175,919	Intergenic	G	C	0.004	0.00009	1.72	0.29	4.1 × 10^–9^
	2		–	82,474,946	–	G	A	0.004	–	1.72	0.29	4.1 × 10^–9^
	2		rs576414992	82,654,697	Intergenic	G	C	0.004	0.00006	1.72	0.29	4.1 × 10^–9^
	2	*LOC105374831*	rs73941786	82,835,859	Intron	C	A	0.004	0.00833	1.72	0.29	4.1 × 10^–9^
	2	*LOC105374834*	rs190806297	83,537,814	Intron	A	G	0.004	0.00004	1.72	0.29	4.1 × 10^–9^
	2		rs140340075	83,662,388	Intergenic	C	A	0.004	–	1.72	0.29	4.1 × 10^–9^
	2		rs188113595	83,930,989	Intergenic	T	C	0.004	0.00144	1.72	0.29	4.1 × 10^–9^
	2		rs573444805	84,150,305	Intergenic	G	C	0.004	0.00004	1.72	0.29	4.1 × 10^–9^
	2		rs548781149	84,159,599	Intergenic	T	C	0.004	0.00004	1.72	0.29	4.1 × 10^–9^
	2		–	84,341,726	–	C	C	0.004	–	1.72	0.29	4.1 × 10^–9^
	2		–	84,464,745	–	T	G	0.004	–	1.72	0.29	4.1 × 10^–9^
	2		–	84,535,416	–	C	C	0.004	–	1.72	0.29	4.1 × 10^–9^
Red cell distribution width	9	*SVEP1*	rs189173017	110,549,951 110,453,573	Intron	G	A	0.004	0.00294	− 1.90	0.30	8.4 × 10^–10^
Red cell distribution width	9	*LPAR1*	rs145221983	110,885,694	Intron	G	C	0.002	0.00178	− 2.64	0.39	3.9 × 10^–11^
platelet distribution width	20	*APCDD1L*	rs73183273	58,478,356	Intron	A	C	0.007	0.00483	1.44	0.23	4.1 × 10^–9^
**MANOLIS**												
Red blood cell count	15		rs1320751535	101,913,651	Intergenic	G	A	0.009	0.00002	1.52	0.23	6.2 × 10^–10^

Betas are reported in units of standard deviation of the traits. The lead SNP for the locus on chromosome 2 is represented by 16 variants in perfect linkage disequilibrium covering an area of 3 Mb.*A0 is the reference allele and A1 is the effect allele for which the allele frequency (AF) is reported in the current sample and in TopMed.

The G-allele (minor allele frequency (MAF) = 0.009) of rs1320751535 at 15q26 was associated with an increase in red blood cell count by 1.52 units of standard deviation error (SE = 0.23, *p* = 6.2 × 10^–10^) in MANOLIS. This single nucleotide variant (SNV) is extremely rare in reference samples. It is not observed in the 1000 Genomes Project data and is carried by 2 out of 125,568 individuals in TopMed^18^. Of note, the credible set in this region, a group of variants that is likely to contain the causal one, included only two markers, neither of which has been reported in previous GWAS for blood traits.

In the Pomak population, we identified four novel loci, including an association at 2p11.2 with increased white blood cell count (WBC) (beta = 1.72, SE = 0.29, *p* value = 4.1 × 10^–9^). The locus contains 16 highly associated variants in perfect linkage disequilibrium that span a region of 3 Mb (Supplementary Material, Supplementary Table 2). One of the seven variants in the credible set was associated with WBC at nominal significance in a large cosmopolitan GWAS^1^ (Supplementary Material, Supplementary Table 3). There was also a novel association with platelet distribution width (PDW) at 20q13.32. Variant rs73183273 (beta = 1.44, SE = 0.23, *p* value = 4.1 × 10^–9^) is located within an intron of the APC down-regulated 1 like (*APCDD1L*) gene. We also identified an association of rs189173017 with red cell distribution width (RDW) (beta = - 1.90, SE = 0.30, *p* value = 8.4 × 10^–10^). This variant is located in an intron of sushi, von Willebrand factor type A, EGF and pentraxin domain containing 1 (*SVEP1*) at 9q31.3 and has a Regulome Database (RDB) score of 5, indicating regulatory function (Supplementary Material, Supplementary Table 2). Variants in the credible set, including rs201343203 intronic to *FSD1L* and rs186868542 intronic to *TMEM38B*, overlap with enhancer sites that are active in blood. In a previous study, an association with platelet distribution width has been reported for another variant in *SVEP1*: missense mutation rs6175193^1^, but not with RDW. There was another novel association on chromosome 9. The rare G-allele of a variant at 9q31.3, rs145221983, had a large effect on RDW with a beta of − 2.62 (SE = 0.39, *p* value = 3.9 × 10^–11^) in the Pomak population. It is intronic to lysophosphatidic acid receptor 1 (*LPAR1*). The encoded membrane protein belongs to a group known as endothelial differentiation gene receptors which mediate platelet aggregation. Common variants in *LPAR1* have previously been linked to haematological traits^1^.

We replicated several previously-reported associations^1^ at genome-wide significance, mostly with platelet traits. Variant rs11553699 at 12q24.31 was associated with mean platelet volume (beta = 0.39, SE = 0.06, *p* value = 4.9 × 10^–11^) and platelet distribution width (beta = 0.39, SE = 0.06, *p* value = 2.3 × 10^–10^) in MANOLIS, and rs1354034 at 3p14.3 and rs342293 at 7q22.3 with large platelet distribution ratio (beta = 0.23, SE = 0.037, *p* value = 7.0 × 10^–10^ and beta = 0.22, SE = 0.04, *p* value = 1.4 × 10^–9^, respectively), mean platelet volume (beta = 0.24, SE = 0.04, *p* value = 1.4 × 10^–10^ and beta = 0.23, SE = 0.04, *p* value = 3.8 × 10^–10^, respectively) and platelet distribution width (beta = 0.23, SE = 0.04, *p* value = 2.1 × 10^–9^ and beta = 0.22, SE = 0.04, *p* value = 3.9 × 10^–9^, respectively) in the Pomak population (Supplementary Table 4).

### HBB mutations

**Figure 1:**
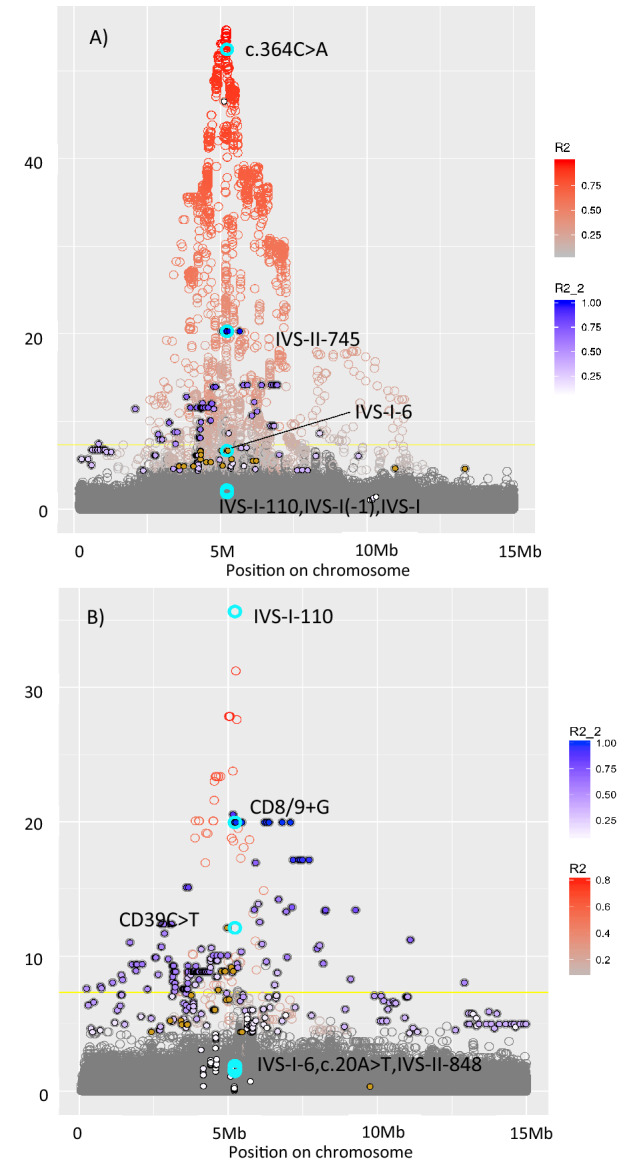
Regional association plot for variants located between 0 and 15 Mb on chromosome 11. Each circle represents a genetic variant. They are arranged on the x-axis by their location. The y-axis shows the p-value for their association with red cell distribution width in (**A**) Pomak and (**B**) MANOLIS. Pathogenic *HBB* mutations are highlighted in turquoise and labelled. The colouring of the circle (R2) indicates the strength of linkage disequilibrium (LD) with the most strongly associated *HBB* mutation, c.364C>A in MANOLIS and IVS-I-110 in Pomak. The blue filling of points (R2_2) indicates the strength of LD with the second most strongly associated *HBB* mutation, IVS-II-745 in MANOLIS and CD8/9+G in Pomak. The brown filling of the circle indicates variants in LD with the third most strongly associated *HBB* mutation, IVS-I-6 in MANOLIS and CD39C>T in Pomak.

Across the entire genome, a region on chromosome 11 displayed the strongest association with all measured red blood cell traits, except haematocrit (HCT) in Pomak and mean corpuscular haemoglobin concentration (MCHC) in MANOLIS (Supplementary Material, Supplementary Fig. 1). Conditional analyses demonstrated that the chr11 peak in the Pomak population could be explained by the three most frequent mutations in the HBB gene, c.364C>A, IVS-II-745 and IVS-I-6 (Fig. 1). The same was true for MANOLIS, again with three independent signals from *HBB* mutations, IVS-I-110, CD8/9+G and CD39C>T. We identified all mutations in the HBB gene that have previously been classified as pathogenic, as described in the methods section. In both groups, we observed high proportions of carriers. A total of 99 individuals in MANOLIS (6.8% of the individuals) carried a pathogenic *HBB* mutation and 162 individuals in the Pomak population (10.0% of the individuals). Different mutation spectra were observed in the two populations (Table 2). There were ten mutations overall, six observed in MANOLIS and six in the Pomak population. Consequences included missense, splice site, splice donor, stop gained and frameshift mutations.

**Table 2:** Pathogenic *HBB* mutations in Pomak and MANOLIS and their associations with red cell distribution width.

Mutation	rs-id	Consequence	type	Position	Allele frequency	N carriers	Beta*	SE	P-value*
**Pomak (N = 1617)**									
HbO-Arab c.364G>A (p.Glu122Lys)	rs33946267	Missense	HbO	5,225,678	0.044	139	− 1.35	0.085	1.3 × 10^–51^
IVS-II-745c.316-106C> G	rs34690599	Splice site	β +	5,225,832	0.004	14	− 2.79	0.291	4.7 × 10^–21^
IVS-I-110 c.93-21G>A	rs35004220	Splice site	β +	5,226,820	0.0003	1	− 2.59	0.949	0.006
IVS-I-6 c.92 + 6T>C	rs35724775	Splice site	β +	5,226,924	0.002	7	− 1.99	0.383	2.3 × 10^–7^
IVS-I-1 c.92+1G>A	rs33971440	Splice donor	β0	5,226,929	0.0003	1	− 2.39	0.968	0.014
IVS-I (-1) c.92G>A (p.Arg31Lys)	rs33960103	Missense	β0	5,226,930	0.0003	1	− 2.40	0.908	0.008
**MANOLIS (N = 1457)**									
IVS-II-848 c.316-3C>A	rs33913413	Splice site	β +	5,225,729	0.0003	1	− 2.12	0.991	0.033
CD39 c.118C>T (p.Gln40Ter)	rs11549407	Stop gained	β0	5,226,774	0.005	13	− 2.06	0.285	7.4 × 10^–13^
IVS-I-110 c.93-21G>A	rs35004220	splice site	β +	5,226,820	0.015	44	− 2.16	0.166	3.2 × 10^–36^
IVS-I-6 c.92 + 6T>C	rs35724775	splice site	β +	5,226,924	0.001	3	− 1.40	0.562	0.013
CD8/9 + G c.27dupG (p.Ser10Valfs*14)	rs35699606	frameshift	β0	5,226,995	0.009	23	− 2.35	0.247	1.1 × 10^–20^
c.20A>T (p.Glu7Val)	rs334	missense	HbS	5,227,002	0.004	15	− 0.79	0.342	0.021

* Regression coefficient beta and p value for the association of the variant with red cell distribution width in units of standard deviation.

### Mutation spectrum in the Pomak population

The missense variant c.364C>A (rs33946267), also known as HbO-Arab^19^, was the most common pathogenic mutation in *HBB* with 139 carriers and an allele frequency of 4.4%. While no carriers of this mutation were observed in MANOLIS, 85% of HBB carriers in the Pomak group had the c.364C>A mutation. Variants in linkage disequilibrium (LD) with c.364C>A in the Pomak population spanned a range of almost 10 Mb from approximately 2,500,000 to 11,000,000 (Fig. 1).

### Mutation spectrum in MANOLIS

IVS-I-110 (G>A) was also present in MANOLIS, where it was the most common *HBB* mutation with 44 carriers of this mutation which represent 44% of all carriers in MANOLIS. Additional mutations in MANOLIS included (Table 2): CD39 (C>T) (rs11549407), CD8/9+G (rs35699606) and the sickle cell HbS mutation c.20A>T (rs334). CD8/9+G in MANOLIS was located on a haplotype ranging from 0 to 15,000,000.

### Characterisation of phenotypic effects of HBB mutations

To assess the effects on haematological traits, we grouped *HBB* mutations into those that either reduce (b+) or abolish (b0) expression of beta-globin (see Table 2 for list of mutations) and analysed c.364C>A (HbO-Arab) in Pomak and sickle cell HbS c.20A>T (rs334) in MANOLIS separately. Carriers of thalassemia variants were characterised by microcytosis (decreased mean corpuscular volume), hypochromia (decreased mean corpuscular haemoglobin), mild anaemia (decreased haemoglobin) as well as decreased mean corpuscular haemoglobin concentration, haematocrit, red cell distribution width and increased red blood cell count (Table 3). We also observed increased platelet distribution width and large platelet distribution ratio in Hb0 carriers in MANOLIS. In line with previous reports^20^, c.364C>A (HbO-Arab) led to increased mean corpuscular haemoglobin concentration while thalassemia variants were linked to decreased values. The effects of the HbO-Arab mutation extended beyond red blood cell traits: for 12 out of 15 measured haematological traits, we observed significant differences after Bonferroni correction (*p* < 0.0013). These included associations with platelet traits: increased platelet distribution width (beta = 0.62, SE = 0.18, *p* value = 4.2 × 10^–4^), mean platelet volume (beta = 0.34, SE = 0.09, *p* value = 1.6 × 10^–4^) and large platelet distribution ratio (beta = 2.77, SE = 0.70, *p* value = 7.5 × 10^–5^). We also observed an increased white blood cell count (beta = 1.25, SE = 0.17, *p* value = 1.6 × 10^–13^) for HbO-Arab carriers and consistent effects on lymphocyte (beta = 0.36, SE = 0.06, *p* value = 1.3 × 10^–8^) and neutrophil count (beta = 0.77, SE = 0.14, *p* value = 9.8 × 10^–8^).

**Table 3:** Differences in haematological, cardiometabolic and anthropometrics traits between carriers and non-carriers of *HBB* mutations. width RDW-SD in Pomak, RDW-a in Manolis.

Trait	Unit	Pomak				MANOLIS			
		Type	Beta	SE	*P* value	Type	Beta	SE	*P *value
Red cell distribution	fl	b+	− 10.45	0.74	**< 2e−16**	b+	− 16.78	0.90	**< 2e−16**
		b0	− 12.06	2.28	**1.4e−07**	b0	− 17.28	0.97	**< 2e−16**
		HbO	− 6.42	0.30	** < 2e−16**	HbS	− 6.92	1.81	**1.4E−04**
Red blood cell count	10^12/l	b+	1.02	0.08	** < 2e−16**	b+	0.88	0.08	** < 2e−16**
		b0	0.82	0.26	**1.3E−03**	b0	1.03	0.08	** < 2e−16**
		HbO	0.30	0.03	** < 2e−16**	HbS	0.15	0.14	0.30
Haemoglobin	g/l	b+	− 1.92	0.28	**4.5e−12**	b+	− 1.85	0.20	** < 2e−16**
		b0	− 2.70	0.84	1.4e−03	b0	− 2.15	0.21	** < 2e−16**
		HbO	0.20	0.11	0.07	HbS	− 0.68	0.37	0.07
Haematocrit	%	b+	− 2.82	0.71	**7.4e−05**	b+	− 4.70	0.59	**3.7E−15**
		b0	− 5.71	2.17	8.7e−03	b0	− 5.50	0.62	** < 2e−16**
		HbO	− 1.09	0.29	**1.3e−04**	HbS	− 2.39	1.09	0.03
Mean corpuscular volume	fl	b+	− 20.35	1.06	** < 2e−16**	b+	− 21.42	0.83	** < 2e−16**
		b0	− 24.49	3.27	**1.1e−13**	b0	− 24.20	0.87	** < 2e−16**
		HbO	− 7.43	0.43	** < 2e−16**	HbS	− 7.21	1.54	**3.2E−06**
Mean corpuscular haemoglobin	pg	b+	− 8.47	0.46	** < 2e−16**	b+	− 7.79	0.31	** < 2e−16**
		b0	− 9.67	1.42	**1.6e−11**	b0	− 8.77	0.33	** < 2e−16**
		HbO	− 1.34	0.19	**1.3e−12**	HbS	− 2.18	0.58	**1.9E−04**
Mean corpuscular haemoglobin concentration	g/dl	b+	− 2.51	0.25	** < 2e−16**	b+	− 0.64	0.15	**2.2E−05**
		b0	− 2.40	0.77	1.8e−03	b0	− 0.67	0.16	**2.9E−05**
		HbO	1.39	0.10	** < 2e−16**	HbS	0.41	0.28	0.14
Platelet count	10^9/l	b+	26.81	12.64	0.03	b+	6.38	8.73	0.46
		b0	− 7.31	38.76	0.85	b0	− 8.64	9.19	0.35
		HbO	5.53	5.11	0.28	HbS	− 41.88	16.18	9.8E−03
Platelet distribution width	fl	b+	0.21	0.46	0.64	b+	0.42	0.19	0.03
		b0	0.33	1.87	0.86	b0	0.87	0.21	**5.4E−05**
		HbO	0.62	0.18	**4.2e−04**	HbS	− 0.56	0.40	0.16
Mean platelet volume	fl	b+	− 0.22	0.24	0.35	b+	0.18	0.14	0.21
		b0	− 0.71	0.97	0.46	b0	0.44	0.15	3.1E−03
		HbO	0.34	0.09	**1.6e−04**	HbS	− 0.36	0.26	0.16
Plateletcrit	%	b+				b+	0.01	0.01	0.08
		b0				b0	0.01	0.01	0.31
		HbO				HbS	− 0.05	0.01	**5.8E−04**
Large platelet distribution ratio	%	b+	− 0.45	1.82	0.80	b+	2.24	0.92	0.02
		b0	− 3.30	7.45	0.66	b0	4.70	1.00	**2.9E−06**
		HbO	2.77	0.70	**7.5e−05**	HbS	− 2.74	1.86	0.14
Granulocyte count	10^9/l	b+				b+	0.38	0.25	0.12
		b0				b0	0.79	0.27	3.4E−03
		HbO				HbS	− 0.35	0.50	0.49
White blood cell count	10^9/l	b+	0.53	0.42	0.21	b+	0.67	0.30	0.03
		b0	0.70	1.28	0.58	b0	0.87	0.32	6.5E−03
		HbO	1.25	0.17	**1.6e−13**	HbS	− 0.76	0.56	0.18
Lymphocyte count	10^9/l	b+	0.38	0.16	0.01	b+	0.22	0.12	0.05
		b0	1.29	0.48	7.2e−03	b0	− 0.03	0.12	0.78
		HbO	0.36	0.06	**1.3e−08**	HbS	− 0.38	0.22	0.08
Neutrophil count	10^3/L	b+	− 0.27	0.39	0.48	b+			
		b0	− 0.54	1.03	0.60	b0			
		HbO	0.77	0.14	**9.8e−08**	HbS			
Mixed cell count	10^3/L	b+	0.05	0.07	0.47	b+	0.02	0.02	0.34
		b0	− 0.05	0.18	0.76	b0	0.02	0.03	0.49
		HbO	0.03	0.02	0.19	HbS	− 0.04	0.05	0.44
C-reactive protein	nmol/L	b+	− 1.32	14.12	0.93	b+	3.08	16.76	0.85
		b0	− 8.13	40.97	0.84	b0	3.83	19.69	0.85
		HbO	0.10	5.85	0.99	HbS	− 2.16	28.97	0.94
Ferritin	pmol/L	b+	45.05	41.42	0.28	b+	12.46	41.02	0.76
		b0	302.18	127.04	0.02	b0	91.95	46.69	0.05
		HbO	24.78	17.07	0.15	HbS	− 66.81	71.68	0.35
Iron	mmol/L	b+	− 1.14	1.59	0.47	b+	0.52	0.86	0.54
		b0	3.86	4.86	0.43	b0	1.59	0.98	0.10
		HbO	1.57	0.65	0.02	HbS	− 2.70	1.50	0.07
Glucose	mmol/l	b+	0.01	0.40	0.99	b+	− 0.31	0.28	0.26
		b0	− 1.61	1.21	0.19	b0	0.38	0.31	0.23
		HbO	− 0.07	0.16	0.66	HbS	0.98	0.48	0.04
Insulin	pmol/L	b+	− 8.46	28.31	0.77	b+	− 11.73	22.27	0.60
		b0	− 38.26	86.83	0.66	b0	92.40	25.09	0.00
		HbO	− 11.76	11.67	0.31	HbS	37.28	38.51	0.33
High-density lipoprotein	mmol/L	b+	− 0.21	0.07	3.2e−03	b+	− 0.04	0.05	0.47
		b0	− 0.25	0.22	0.25	b0	− 0.10	0.06	0.09
		HbO	− 0.05	0.03	0.12	HbS	− 0.12	0.09	0.15
Low-density lipoprotein	mmol/L	b+	− 0.14	0.21	0.50	b+	− 0.43	0.14	2.5E−03
		b0	1.21	0.64	0.06	b0	− 0.44	0.16	6.4E−03
		HbO	0.01	0.09	0.90	HbS	0.02	0.25	0.93
Triglycerides	mmol/L	b+	− 0.06	0.21	0.79	b+	− 0.16	0.17	0.35
		b0	0.02	0.63	0.98	b0	− 0.25	0.19	0.19
		HbO	0.01	0.08	0.89	HbS	0.25	0.30	0.41
Total cholesterol	mmol/L	b+	− 0.38	0.23	0.11	b+	− 0.53	0.16	**9.6E−04**
		b0	0.96	0.72	0.18	b0	− 0.65	0.18	**4.3E−04**
		HbO	− 0.03	0.10	0.77	HbS	0.01	0.28	0.97
Thyroid stimulating hormone	uIU/ml	b+	− 0.03	0.87	0.97	b+	− 0.23	0.49	0.64
		b0	0.75	3.36	0.82	b0	− 0.54	0.58	0.35
		HbO	− 0.19	0.32	0.56	HbS	− 0.20	0.87	0.82
Free thyroxine	ng/dl	b+	− 0.12	0.05	0.01	b+	0.07	0.03	0.03
		b0	− 0.11	0.19	0.56	b0	0.00	0.04	0.99
		HbO	0.05	0.02	0.01	HbS	0.01	0.06	0.85
Osteocalcin	ng/ml	b+	2.18	3.20	0.50	b+	2.19	1.34	0.10
		b0	9.15	10.56	0.39	b0	− 1.31	1.59	0.41
		HbO	1.07	1.18	0.36	HbS	− 2.39	2.31	0.30
Bilirubin	mg/dl	b+	0.08	0.02	**1.2e−04**	b+	0.04	0.01	2.2E−03
		b0	0.07	0.08	0.39	b0	0.05	0.01	**8.8E−04**
		HbO	0.04	0.01	**2.8e−07**	HbS	− 0.02	0.02	0.39
Alanine aminotransferase	iu/l	b+	− 1.15	1.84	0.53	b+	− 5.42	1.79	2.5E−03
		b0	− 4.95	7.10	0.49	b0	− 3.82	2.13	0.07
		HbO	0.62	0.67	0.36	HbS	− 4.37	3.10	0.16
Gamma-glutamyl transferase	iu/l	b+	4.49	3.95	0.26	b+	− 5.76	3.24	0.08
		b0	− 6.96	15.22	0.65	b0	− 0.80	3.86	0.84
		HbO	2.53	1.44	0.08	HbS	− 6.24	5.79	0.28
Leptin	ng/ml	b+				b+	− 1.79	3.51	0.61
		b0				b0	− 7.42	4.52	0.10
		HbO				HbS	13.75	6.35	0.03
Adiponectin	ug/ml	b+				b+	− 0.54	0.49	0.27
		b0				b0	− 0.77	0.54	0.15
		HbO				HbS	− 2.31	0.95	0.01
Weight	kg	b+	3.94	3.29	0.23	b+	− 0.71	2.22	0.75
		b0	− 4.97	10.09	0.62	b0	1.34	2.50	0.59
		HbO	0.12	1.34	0.93	HbS	6.91	3.67	0.06
Height	cm	b+	0.66	1.54	0.67	b+	− 0.41	0.09	**7.8E−06**
		b0	5.30	4.60	0.25	b0	− 2.01	1.12	0.07
		HbO	− 0.53	0.61	0.39	HbS	5.98	1.69	**4.0E−04**
Waist-hip ratio		b+	0.03	0.02	0.11	b+	− 0.01	0.01	0.52
		b0	0.02	0.06	0.70	b0	0.02	0.01	0.13
		HbO	0.01	0.01	0.08	HbS	0.00	0.02	0.85
Body-mass-index		b+	1.65	1.21	0.18	b+	− 0.38	0.82	0.65
		b0	− 3.75	3.62	0.30	b0	1.25	0.92	0.17
		HbO	0.27	0.48	0.57	HbS	0.61	1.37	0.66
Gestation age	Months	b+				b+	− 0.41	0.09	**7.8E−06**
		b0				b0	0.03	0.08	0.66
		HbO				HbS	0.03	0.10	0.72

We grouped *HBB* mutations into those that either reduce (b +) or abolish (b0) expression of beta-globin (see Table 2). We also separated c.364C>A (HbO-Arab) in Pomak and sickle cell HbS in MANOLIS. Associations significant after Bonferroni correction (*p* value < 0.0013) were bolded.

**Figure 2:**
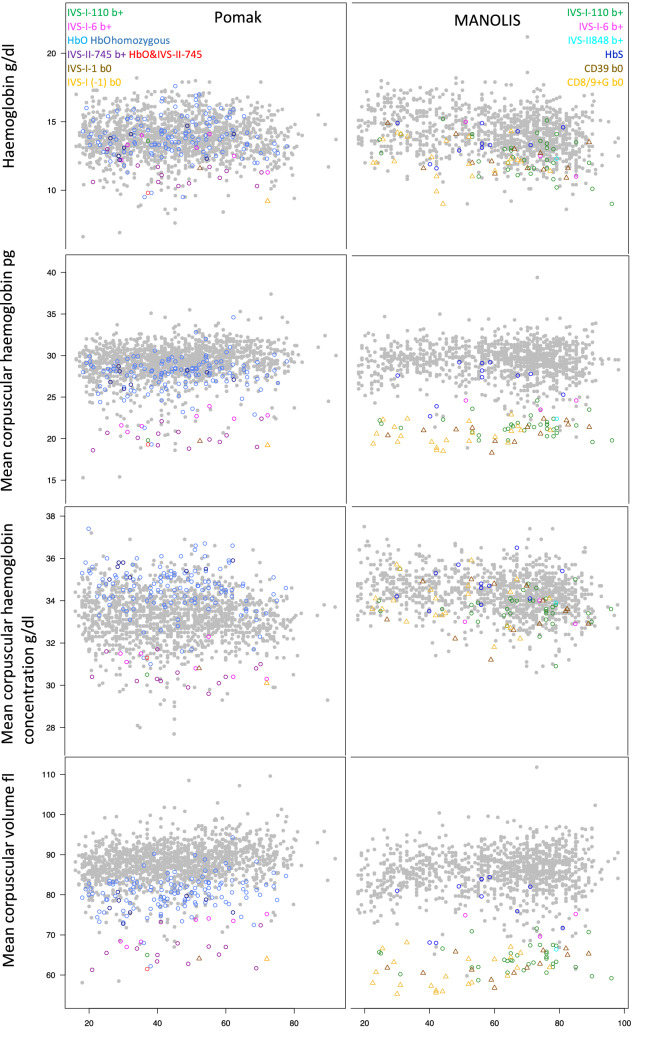
Values for different red cell traits (y-axis) by age (x-axis) for carriers of different *HBB* mutations in Pomak and MANOLIS. Individuals without a detected HBB mutation are shown as grey points. Values for carriers of particular mutations are shown using different colours as indicated on the plot.

We tested whether the effects on red cell traits differ between specific *HBB* mutations. Across the mutational spectrum, there was a clear separation between carriers and non-carriers (Fig. 2), and each mutation was individually associated with red cell traits, such as RDW levels (Table 2). A case-only analysis demonstrated differences between the structural variants and thalassemia mutations (Supplementary Material, Supplementary Table 5). Moreover, in both Pomak and MANOLIS, IVS-I-6 had significantly less severe effects on red cell distribution width, mean corpuscular volume and mean corpuscular haemoglobin compared to the most common b+ mutation in each group.

We also observed effects of *HBB* mutations on several cardiometabolic-related blood biomarkers. Carriers of all *HBB* mutations except HbO had increased bilirubin levels in both populations. In MANOLIS, thalassemia mutation carriers had decreased total cholesterol (beta = − 0.53 and beta = − 0.65 for b+ and b0, respectively) which coincided with decreased values of low-density lipoprotein (LDL) cholesterol (beta = − 0.43 and beta = − 0.44, respectively). Finally, there were differences in other traits in MANOLIS. Carriers of HbS were significantly taller (beta = 5.98, SE = 1.69, p-value = 4.0 × 10^–4^). Earlier gestation age was seen in carriers of b+ mutations (beta = − 0.41, SE = 0.09, *p* value = 7.8 × 10^–6^).

## Discussion

We present a first detailed characterisation of the mutational spectrum of haemoglobin in two isolated Mediterranean populations, HELIC MANOLIS (Crete) and the HELIC Pomak cohort. A large proportion of the variation in the haematological traits can be explained by *HBB* mutations in these populations. We provide the first effect estimates for two large non-clinically ascertained samples. We also demonstrate an important role for rare non-coding variation. Finally, we replicate associations of common variants with smaller effects that have previously been reported for cosmopolitan populations with European ancestry.

We discover novel associations of variants at 15q26, 2p11.2, 20q13.32, and 9q31.3 with red and white blood cell count, platelet and red cell distribution width, respectively. All of these variants are rare and located outside of coding regions, making it difficult to understand the exact mechanisms through which these variants affect blood traits. Several genes in the 9q31.3 region have been associated with abnormal lipid profiles and coronary artery disease, including SVEP1^21^ and ATP Binding Cassette Subfamily A Member 1 gene (*ABCA1*)^22,23^. Links between haematological traits and lipid profiles have been previously demonstrated^24^. Another variant at the locus with likely regulatory function, rs201343203, is intronic to Fibronectin Type III and SPRY Domain Containing 1 Like (FSD1L), a gene that has been previously linked to red cell distribution width^25^.

For the PDW-associated variants at 20q13.32, results from chromatin interaction experiments^26^ further implicate *APCDD1L* as a likely target gene. An important paralog of this gene (APCDD1) is a negative regulator of the Wnt signaling pathway that is involved in the regulation of platelet function^27^. Chromatin immunoprecipitation sequencing experiments have also shown that the Histone-lysin N-methyltransferase SETDB1 protein binds in the lead SNP in this region. Furthermore, female heterozygous mutant mice have abnormal peripheral blood lymphocytes data^28^. Further investigation is required to fully elucidate the underlying mechanisms of these novel associations.

In each of the two populations studied here, moderate to large effects are observed, with alleles conferring effects of 1.44–2.63 units of standard deviation. This contrasts with other studies. For example, a study based on whole-genome sequencing of 3,781 individuals from a cosmopolitan European population did not discover any novel associations with blood traits^29^. We hypothesize that the observed enrichment of novel haematological association of rare variants is a consequence of population history^30^. Genetic drift due to the founder event in these isolated groups may have resulted in increased allele frequencies for the associated variants, which provides better statistical power for discovery of rare variants with large impact^16^. In fact, most of the lead variants at the novel loci have risen in frequency compared to large reference populations. For example, the rare allele of rs1320751535 at chromosome 15 is only carried by two individuals in TopMed (MAF = 0.000016) but has a frequency of almost 1% in MANOLIS. Limitations of genetic association studies include the possibility of false positive associations. Relative to some array-based genotyping efforts, our sample size was smaller. To fully confirm these novel associations, replication studies would be warranted. However, the low frequency of these variants in other populations and the need for large-scale sequencing to detect them has prevented replication testing using data from other available studies.

Previous reports of high levels of mutational diversity were limited to cosmopolitan populations. We found six different pathogenic *HBB* mutations in MANOLIS and six in the Pomak group, two of which were seen in both populations. We compared the mutational spectrum observed in the two Greek isolated populations to published data from 3,796 individuals, who were referred to genetic counselling at the NTC from all over Greece^31^. The two most common variants seen in the Greek NTC samples, IVS-I-110G>A (42.1% of carriers) and CD39C>T (18.8% of carriers), were both relatively common in MANOLIS (44% and 13% of carriers, respectively)). However, some of the mutations from the NTC sample, such as IVS-I-1 (G>A) (12.8% of carriers in NTC) and IVS-II-745C>G (6.3%), were not present in MANOLIS. Conversely, the second most frequent variant in MANOLIS, CD8/9+G, was rare in the NTC sample (0.1% of carriers). It should be noted, however, that recruitment for NTC was for symptomatic cases and relatives which may affect the mutation spectrum. Therefore, the carrier frequencies may not be comparable. There were marked differences in frequency patterns between the Pomak population and NTC sample with the top three mutations from NTC sample, IVS-I-110G>A, CD39C>T, and IVS-I-1, either not present or carried by only one individual in the Pomak population. The most common mutation in Pomak, c.364C>A (HbO), was not observed in the NTC sample. It has been previously postulated that this variant originated in the Pomak population^19^.

We provide detailed additional information to characterise the effects of this variant based on data of heterozygous and homozygous carriers. Firstly, we confirm a high allele frequency of 4.4% in the Pomak sample. In line with previous reports, we observe decreased levels of mean corpuscular volume but increases in mean corpuscular haemoglobin concentration^20,32^. This can be explained by the strong positive charge of the HbO molecule which results in their accumulation below the inner surface of the negatively charged erythrocyte membrane, leading to more space that can be filled with haemoglobin as well as denser, more spherical cells^33^. However, in discordance with previous research^20,32^, we do not find statistically significant differences between heterozygotes and homozygotes for these traits. Moreover, we observe novel effects of HbO on white blood cell traits and platelets, including increased platelet distribution width, mean platelet volume, large platelet distribution ratio, as well as white blood cell, lymphocyte and neutrophil count.

The isolated Cretan population has low levels of cardiometabolic complications despite exposure to risk factors such as obesity^15^. In line with previous reports^34–36^, we found decreased levels of total and LDL-cholesterol in carriers of thalassemia variants in MANOLIS. This has previously been linked to a decreased risk of atherosclerotic cardiovascular disease^37–42^. In MANOLIS we also found an association between the sickle cell HbS mutation with increased height. However, it cannot be ruled out that carrier status tags a specific ancestral group with taller stature. There was also a novel association of b+ mutations with increased risk of being born pre-term.

We show that c.364C>A (HbO-Arab), IVS-II-745 (C>G), CD8/9 + G and IVS-I-110 (G>A) are located on a very large haplotype each, extending over up to 15 Mb, a range that has previously only been reported for the major histocompatibility complex (MHC) region. This pattern likely represents a trace of the natural history of haemoglobin disorders. Positive natural selection can lead to increased linkage disequilibrium^43^. There is strong evidence that heterozygous carrier status of certain haemoglobin mutations provides a protective effect against malaria^9^ and Greece is one of the regions with a history of long-standing endemic malaria^10^.

In conclusion, whole-genome sequencing enabled a detailed characterisation of the spectrum of mutations, providing important insights into the allelic architecture of medically-relevant haematological traits. This can provide important guidance for mutation screening in these regions. Future research should extend this work to other populations with a high prevalence of haemoglobin disorders.

## Materials and methods

### Samples

The HELIC cohorts (https://www.helmholtz-muenchen.de/itg/projects-and-cohorts/helic/index.html) have previously been described in detail^15–17^. Briefly, MANOLIS includes individuals from the mountainous Mylopotamos villages on Crete. Individuals for the Pomak cohort were recruited at the Pomak villages, a set of mountainous villages in the North of Greece. Genetic isolatedness has been demonstrated for both cohorts^16^. A wide range of phenotypic information was collected including anthropometric and biometric measurements, biochemical and haematological blood measures, medical history, demographic, socioeconomic and lifestyle information. All participants provided written informed consent. Ethical approval was obtained from the Harokopio University Bioethics Committee. All methods were performed in accordance with the relevant guidelines and regulations.

### Phenotype data

The distribution of each haematological trait was assessed. For those not sufficiently approximating a normal distribution, log- or rank-based inverse normal transformation was applied to phenotype measures and outliers were excluded (Supplementary Material, Supplementary Table 1). Values were adjusted for sex, age and squared age if any of these were significantly (*p* < 0.05) associated with the trait in a linear regression analysis. Standardised residuals from these analyses were used as outcome for the genome-wide association analysis. For the characterisation of pathogenic mutations in haemoglobin genes, we used un-transformed values as outcomes in the regression analyses to retain interpretability in original units. These analyses were conducted in R v3.4^44^.

### Sequencing

Whole-genome sequencing was carried out for 1,482 samples from MANOLIS and 1,642 samples from Pomak using Illumina’s HiSeqX platform with a target depth of 30x. Processing followed the GATK best practice guideline and has been described in detail elsewhere^45^. Alignment was carried out using BWA mem 0.7.8 using hg38 as the reference. Picard was used to mark duplicates. HaplotypeCaller v.3.5 was used to call genotypes. VQSR was used to for variant quality control (QC) using a tranche threshold of 99.4% for single nucleotide polymorphisms (SNP). For indels, we used the recommended threshold of 1%. We also filtered out 14% of variants with call rates < 99%.

We excluded 25 samples from MANOLIS that failed one or more of the following checks: four samples failed sex checks, eight had low concordance with previous genotyping efforts^17^, eleven were duplicates, twelve samples contaminated. For Pomak a total of 25 samples failed QC: three were duplicates, thirteen were heterozygosity outliers, eight were sex check failures and one was a depth outlier.

### Genome-wide association analyses

The association of genetic variants with each haematological trait was evaluated using a linear mixed model implemented in GEMMA^46^. This approach accounts for relatives in the sample as well as any population substructure. GEMMA was used to estimate the genetic relatedness matrix after filtering for minor allele frequency (MAF) < 0.05, missingness < 1% and linkage disequilibrium (LD)-based pruning. We considered associations of variants with minor allele count of at least 10. To determine the multiple-testing burden, we estimated the effective number of traits by carrying out a principal component analysis for the correlation matrix of traits. The first 10 principal components explained 99% of the variants, therefore the effective number of traits was estimated to be 10. We also accounted for number of variants which resulted in an adjusted p-value threshold of 5 × 10^–9^. Measures of linkage disequilibrium, D’ and r^2^, were calculated using plink^47^.

For each of the previously unreported variants significantly associated with haematological traits, we considered all variants in a ± 500 Kb distance. In order to identify potentially causal variants, we excluded SNPs with a likelihood of being causal of less than 1:100, by comparing the likelihood of each SNP from the association analysis with the one of the most strongly associated SNP^48^. The remaining variants at each locus, henceforth called credible set, were annotated used FUMA^49^, Ensembl including VEP^50^, HaploReg^51^ and Open Targets Genetics^52^ to characterise their putative functional impact.

### HBB mutations

We identified all mutations in the Haemoglobin Subunit Beta (HBB) gene that are classified as pathogenic with a review status of at least one star in the ClinVar data base^53^. These were referenced against the HbVar data bank^12^. The HELIC sequence data were queried for these 83 variants.

In addition to single variant association analyses as described above, we also carried out conditional analyses where we included either the most strongly associated variant or the pathogenic *HBB* variants as covariates in the model.

We used burden testing to evaluate the combined effect of variants in *HBB* and linked regulatory elements on blood traits. We followed the approach outlined in^45^. Briefly, we used an extended SKAT-O model^54^ to account for relatedness or population structure as implemented in MONSTER^55^. Boundaries for *HBB* were extracted from GENCODE v25. We applied eleven different conditions: regions of interest (coding regions only, coding and regulatory regions and regulatory regions only), variant filters (inclusion criteria based on severity of predicted consequence) and weighting schemes.

### Ethics approval and consent to participate

All participants provided written informed consent. Ethical approval was obtained from the Harokopio University Bioethics Committee.

## Supplementary Information

Supplementary Information 1.

Supplementary Information 2.

## Data Availability

The data generated and/or analysed during the current study (i.e. the HELIC genotype and WGS datasets) are availableon the the European Genome-phenome Archive (https://www.ebi.ac.uk/ega/home): EGAD00010000518; EGAD00010000522; EGAD00010000610; EGAD00001001636, EGAD00001001637.

## Abbreviations

APCDD1L: APC down-regulated 1 like
GWAS: Genome-Wide Association Study
HBB: Haemoglobin subunit beta
HELIC: Hellenic isolated cohorts
LD: Linkage disequilibrium
LDL: Low-density lipoprotein
LPAR1: Lysophosphatidic acid receptor 1
MAF: Minor allele frequency
PDW: Platelet distribution width
QC: Quality control
RDW: Red cell distribution width
RDW: Regulome database
SNP: Single nucleotide polymorphism
SNV: Single nucleotide variant
SE: Standard error
SVEP1: Sushi, Von Willebrand Factor Type A, EGF and pentraxin domain containing 1
WBC: White blood cell count
