# Supplementary material for: Insights into the genetic architecture of haematological traits from deep phenotyping and whole-genome sequencing for two Mediterranean isolated populations
Source: Sci Rep. 2022 Jan 21;12:1131. doi: 10.1038/s41598-021-04436-9 (PMC8782863; doi:10.1038/s41598-021-04436-9)
Supplement: Supplementary file 1 — Supplementary Information 1. [file 41598_2021_4436_MOESM1_ESM.docx]

**Insights into the genetic architecture of haematological traits from deep phenotyping and whole-genome sequencing for two Mediterranean isolated populations**

Karoline Kuchenbaecker, PhD^*^, Division of Psychiatry, University College of London, London W1T 7NF, UK; UCL Genetics Institute, University College London, London WC1E 6BT, UK; Department of Human Genetics, Wellcome Sanger Institute, Hinxton, CB10 1SA, UK

Arthur Gilly, PhD, Department of Human Genetics, Wellcome Sanger Institute, Hinxton, CB10 1SA, UK; Institute of Translational Genomics, Helmholtz Zentrum München, German Research Center for Environmental Health, Neuherberg, Germany

Daniel Suveges, PhD, Department of Human Genetics, Wellcome Sanger Institute, Hinxton, CB10 1SA, UK

Lorraine Southam, BSc, Department of Human Genetics, Wellcome Sanger Institute, Hinxton, CB10 1SA, UK; Institute of Translational Genomics, Helmholtz Zentrum München, German Research Center for Environmental Health, Neuherberg, Germany; Wellcome Trust Centre for Human Genetics, University of Oxford, Oxford, OX3 7BN, UK

Olga Giannakopoulou, PhD, Division of Psychiatry, University College of London, London W1T 7NF, UK; UCL Genetics Institute, University College London, London WC1E 6BT, UK

Britt Kilian, MSc, Department of Human Genetics, Wellcome Sanger Institute, Hinxton, CB10 1SA, UK; The Primary Care Unit, Institute of Public Health, University of Cambridge, Box 113 Cambridge Biomedical Campus, Cambridge, CB2 0SR, UK

Emmanouil Tsafantakis, PhD, Anogia Medical Centre, Anogia, 740 51, Greece

Maria Karaleftheri, PhD, Echinos Medical Centre, Echinos, Xanthi 67300, Greece

Aliki-Eleni Farmaki, PhD, Department of Nutrition and Dietetics, School of Health Science and Education, Harokopio University of Athens, Greece; MRC Unit for Lifelong Health and Ageing, Institute of Cardiovascular Science, University College London, London, WC1E 7HB, UK

Deepti Gurdasani, MD, PhD, Department of Human Genetics, Wellcome Sanger Institute, Hinxton, CB10 1SA, UK

Kousik Kundu, PhD, Department of Human Genetics, Wellcome Sanger Institute, Hinxton, CB10 1SA, UK; Department of Haematology, Cambridge Biomedical Campus, University of Cambridge, Long Road, Cambridge, CB2 0PT United Kingdom

Manjinder S. Sandhu, PhD, Department of Medicine, University of Cambridge, Cambridge CB2 0QQ, UK

John Danesh, PhD, Department of Human Genetics, Wellcome Sanger Institute, Hinxton, CB10 1SA, UK; The National Institute for Health Research Blood and Transplant Unit (NIHR BTRU) in Donor Health and Genomics at the University of Cambridge, Strangeways Research Laboratory, Wort’s Causeway, University of Cambridge, Cambridge, CB1 8RN United Kingdom; MRC/BHF Cardiovascular Epidemiology Unit, Department of Public Health and Primary Care, Wort’s Causeway, University of Cambridge, Strangeways Research Laboratory, Cambridge, CB1 8RN United Kingdom

Adam Butterworth, PhD, The National Institute for Health Research Blood and Transplant Unit (NIHR BTRU) in Donor Health and Genomics at the University of Cambridge, Strangeways Research Laboratory, Wort’s Causeway, University of Cambridge, Cambridge, CB1 8RN United Kingdom; MRC/BHF Cardiovascular Epidemiology Unit, Department of Public Health and Primary Care, Wort’s Causeway, University of Cambridge, Strangeways Research Laboratory, Cambridge, CB1 8RN United Kingdom; British Heart Foundation Centre of Excellence, Division of Cardiovascular Medicine, Addenbrooke’s Hospital, Hills Road, Cambridge, CB2 0QQ United Kingdom

Inês Barroso, PhD, Department of Human Genetics, Wellcome Sanger Institute, Hinxton, CB10 1SA, UK; MRC Epidemiology Unit, University of Cambridge, UK

George Dedoussis, PhD, Department of Nutrition and Dietetics, School of Health Science and Education, Harokopio University of Athens, Greece

Eleftheria Zeggini, PhD, Department of Human Genetics, Wellcome Sanger Institute, Hinxton, CB10 1SA, UK; Institute of Translational Genomics, Helmholtz Zentrum München, German Research Center for Environmental Health, Neuherberg, Germany

**Supplementary Material**

*Mutation spectrum in the Pomak population*

The missense variant c.364C>A (rs33946267), also known as HbO-Arab [19], was the most common pathogenic mutation in *HBB* with 139 carriers and an allele frequency of 4.4%. While no carriers of this mutation were observed in MANOLIS, 85% of HBB carriers in the Pomak group had the c.364C>A mutation. Ten individuals were homozygous carriers. In the Pomak population, we also identified 14 carriers of the thalassemia mutation IVS-II-745 (C>G) (rs34690599). This represents 8.6% of *HBB* mutations in the Pomak population, which is comparable to estimates from the National Thalassemia Center (NTC) in Greece (6%) [20]. The other four observed thalassemia variants were rare in this population, with allele frequencies ranging from 0.0003 to 0.002. Seven individuals carried IVS-I-6 (T>C) (rs35724775) which has also been observed in the NTC sample [20]. Each of the following mutations was observed once: IVS-I-1 (G>A) (rs33971440), IVS-I(-1) (G>A) (rs33960103), and IVS-I-110 (G>A) (rs35004220), respectively. IVS-I-1 (G>A) has been reported to be present in 12.8% of HBB mutation carriers in the NTC sample [20].

*Mutation spectrum in MANOLIS*

IVS-I-110 (G>A) was also present in MANOLIS, where it was the most common *HBB* mutation with 44 carriers of this mutation which represent 44% of all carriers in MANOLIS. It has been shown to be the most common beta-thalassemia allele in previous studies in Greece, Cyprus and Turkey [21–23]. Additional mutations in MANOLIS included (Table 2): CD39 (C>T) (rs11549407), CD8/9+G (rs35699606) and the sickle cell HbS mutation c.20A>T (rs334). The stop gain mutation CD39 (C>T) (13% of carriers in MANOLIS) has been found to be the predominant beta-thalassemia allele in Sardinia (95.4%) [24] and the second most frequent in the Greek NTC sample (18.8%) [20]. CD8/9+G (23% of carriers in MANOLIS) was one of the most frequent *HBB* mutations in thalassemia patients from South Asia and the Middle East (e.g., 49% in Pashtuns) [12]. Three individuals carried IVS-I-6 (T>C), which was also present in the Pomak population. IVS-II-848 (C>A) (rs33913413) was carried by only one individual.

*Long-range linkage disequilibrium*

In both populations, the most common mutations in *HBB* were located on haplotypes with long-range LD. Variants in LD with c.364C>A in the Pomak population spanned a range of almost 10Mb from approximately 2,500,000 to 11,000,000 (Figure 1). IVS-II-745 (C>G) was also on a long-range haplotype of about 7Mb. CD8/9+G in MANOLIS was located on a haplotype ranging from 0 to 15,000,000. IVS-I-110 (G>A) was in LD with variants across a region covering 4Mb.

*Burden tests*

We assessed the effect of variants linked to *HBB* using different types of burden tests. The most strongly associated burden implementation with RDW included exonic as well as regulatory variants with a frequency less than 0.05 (p_burden_=4.2x10^-80^ and p_burden_=3.7x10^-51^ in the Pomak population and MANOLIS, respectively). *HBB* was the most strongly associated gene for red blood cell traits.

*Comparison with the UK population*

Results from the two isolates were compared to whole-genome sequencing data across 3,724 individuals from the INTERVAL study. Four different pathogenic mutations were present in *HBB* with one carrier each (Supplementary Material, Supplementary Table 6). One of them overlapped with variants detected in Pomak, c.364C>A (HbO-Arab), and two with variants in MANOLIS, IVS-II-848 (C>A) and CD39 (C>T). Additionally, one individual carried rs63751128 (c.*111A>G). Only CD39 (C>T), also present in MANOLIS, was associated with a nominally significant decrease in RDW. There were no genome-wide associations with RDW on chromosome 11.

**Supplementary Tables**

**Supplementary Table 1.** Variable transformations, outlier exclusions and covariates used for phenotype preparation for genome-wide association analysis

|  |  | **Outlier** |  | **Covariates** | | |
| --- | --- | --- | --- | --- | --- | --- |
| **Trait** | **Short name** | **exclusion** | **Transformation** | **sex** | **age** | **age^2^** |
| white blood cell count | wbc | 4SD* | none | yes | yes | no |
| red blood cell count | rbc | 4SD | none | yes | yes | yes |
| haemoglobin | hgb | 4SD | none | yes | yes | yes |
| hematocrit | hct | 4SD | none | yes | yes | yes |
| mean corpuscular volume | mcv | none | rbin | yes | yes | yes |
| mean corpuscular hemoglobin | mch | none | rbin | yes | yes | yes |
| mean corpuscular hemoglobin concentration | mchc | 4SD | none | yes | yes | no |
| platelet count | plt | 4SD | none | yes | yes | no |
| red cell distribution width | rdw | none | rbin | yes | yes | no |
| platelet distribution width | pdw | none | ln | yes | no | no |
| mean platelet volume | mpv | 4SD | none | yes | yes | no |
| plateletcrit | pct | 4SD | none | yes | yes | no |
| large platelet distribution ratio | lpcr | 4SD | none | yes | no | no |
| granulocyte count | gran | 4SD | ln | yes | yes | yes |
| lymphocyte count | lym | <0.5 | ln | yes | yes | yes |
| neutrophil count | neut | <0.23 | ln | yes | yes | yes |
| mixed cell count | mid/mxd | >1.1 | none | yes | yes | yes |

* values that were more than 4 standard deviations (SD) larger or smaller than the mean

**Supplementary Table 2.** Functional annotation of previously unreported variants that were significantly associated with haematological traits and the rest of variants in the corresponding credible sets.

| **Credible set** | **rsID** | **Chr** | **Position** | **Nearest gene** | **Distance** | **Function** | **CADD** | **RDB** | **minChrState** | **commonChrState** | **Enhancer** |
| --- | --- | --- | --- | --- | --- | --- | --- | --- | --- | --- | --- |
| Pomak - WBC | - | 2 | 81484692 | - | - | - | - | - | - | - | - |
|  | - | 2 | 81745516 | - | - | - | - | - | - | - | - |
|  | - | 2 | 81759339 | - | - | - | - | - | - | - | - |
|  | - | 2 | 81954850 | - | - | - | - | - | - | - | - |
|  | - | 2 | 82175919 | - | - | - | - | - | - | - | - |
|  | - | 2 | 82474946 | - | - | - | - | - | - | - | - |
|  | - | 2 | 82654697 | - | - | - | - | - | - | - | - |
|  | rs73941786 | 2 | 82835859 | AC098817.3 | 16594 | intergenic | 0.322 | 0.41041 | 5 | 15 | - |
|  | rs190806297 | 2 | 83537814 | AC010744.1 | 14311 | intergenic | 6.931 | 0.32023 | 9 | 15 | - |
|  | rs140340075 | 2 | 83662388 | - | - | - | - | 0.66931 | - | - | - |
|  | rs188113595 | 2 | 83930989 | AC104395.1 | 52435 | intergenic | 1.752 | 0.13454 | 14 | 15 | - |
|  | - | 2 | 84150305 | - | - | - | - | - | - | - | - |
|  | - | 2 | 84159599 | - | - | - | - | - | - | - | - |
|  | - | 2 | 84341726 | - | - | - | - | - | - | - | - |
|  | - | 2 | 84464745 | - | - | - | - | - | - | - | - |
|  | - | 2 | 84535416 | - | - | - | - | - | - | - | - |
|  | - | 2 | 82642204 | - | - | - | - | - | - | - | - |
|  | - | 2 | 82318977 | - | - | - | - | - | - | - | - |
| Pomak - RDW | rs145221983 | 9 | 110885694 | LPAR1 | 0 | intronic | - | 0.96117 | - | - | - |
|  | - | 9 | 111255116 | - | - | - | - | - | - | - | - |
|  | - | 9 | 111297888 | - | - | - | - | - | - | - | - |
|  | - | 9 | 111369858 | - | - | - | - | - | - | - | - |
|  | - | 9 | 111775585 | - | - | - | - | - | - | - | - |
|  | rs182113470 | 9 | 111881162 | UGCG | 15603 | intergenic | 9.204 | 0.18412 | 5 | 15 | - |
|  | - | 9 | 112005782 | - | - | - | - | - | - | - | - |
|  | - | 9 | 112103327 | - | - | - | - | - | - | - | - |
|  | rs138523839 | 9 | 112122739 | SUSD1 | 0 | intronic | - | 0.52984 | - | - | - |
|  | - | 9 | 112317999 | - | - | - | - | - | - | - | - |
|  | - | 9 | 112319793 | - | - | - | - | - | - | - | - |
|  | rs77635713 | 9 | 106600661 | RP11-308N19.1 | 0 | ncRNA_intronic | 11.43 | 0.13454 | 9 | 15 | - |
|  | rs74462705 | 9 | 106627773 | RP11-308N19.4 | 0 | ncRNA_intronic | 0.253 | 0.00545 | 5 | 15 | - |
|  | rs111665332 | 9 | 106919659 | ZNF462 | 0 | intronic | - | 0.13454 | - | - | - |
|  | - | 9 | 107190792 | - | - | - | - | - | - | - | - |
|  | - | 9 | 107196770 | - | - | - | - | - | - | - | - |
|  | rs117587268 | 9 | 107332803 | - | - | - | - | 0.58955 | - | - | - |
|  | - | 9 | 107571052 | - | - | - | - | - | - | - | - |
|  | - | 9 | 108623836 | - | - | - | - | - | - | - | - |
|  | - | 9 | 108880384 | - | - | - | - | - | - | - | - |
|  | - | 9 | 109419994 | - | - | - | - | - | - | - | - |
|  | - | 9 | 109533768 | - | - | - | - | - | - | - | - |
|  | - | 9 | 109549645 | - | - | - | - | - | - | - | - |
|  | rs138963580 | 9 | 109592925 | PALM2 | 47862 | intergenic | 1.102 | 0.13454 | 5 | 15 | - |
|  | - | 9 | 109723269 | - | - | - | - | - | - | - | - |
|  | - | 9 | 109725304 | - | - | - | - | - | - | - | - |
|  | rs141803223 | 9 | 109874774 | PALM2 | 0 | intronic | - | 0.00167 | - | - | - |
|  | - | 9 | 110077259 | - | - | - | - | - | - | - | - |
|  | - | 9 | 110120344 | - | - | - | - | - | - | - | - |
|  | - | 9 | 110234106 | - | - | - | - | - | - | - | - |
|  | - | 9 | 110495617 | - | - | - | - | - | - | - | - |
|  | rs189173017 | 9 | 110453573 | SVEP1 | 0 | intronic | 4.766 | 0.15676 | 4 | 15 | - |
|  | rs201551854 | 9 | 110423173 | SVEP1 | 0 | intronic | - | 1.0 | - | - | - |
|  | - | 9 | 111362114 | - | - | - | - | - | - | - | - |
|  | - | 9 | 111383838 | - | - | - | - | - | - | - | - |
|  | - | 9 | 111199635 | - | - | - | - | - | - | - | - |
|  | - | 9 | 107250353 | - | - | - | - | - | - | - | - |
|  | - | 9 | 85709292 | - | - | - | - | - | - | - | - |
|  | - | 9 | 85967943 | - | - | - | - | - | - | - | - |
|  | - | 9 | 86306598 | - | - | - | - | - | - | - | - |
|  | - | 9 | 96719475 | - | - | - | - | - | - | - | - |
|  | - | 9 | 96868742 | - | - | - | - | - | - | - | - |
|  | - | 9 | 96958774 | - | - | - | - | - | - | - | - |
|  | - | 9 | 97086431 | - | - | - | - | - | - | - | - |
|  | - | 9 | 97289904 | - | - | - | - | - | - | - | - |
|  | - | 9 | 97304495 | - | - | - | - | - | - | - | - |
|  | - | 9 | 97494930 | - | - | - | - | - | - | - | - |
|  | - | 9 | 97635938 | - | - | - | - | - | - | - | - |
|  | - | 9 | 97694003 | - | - | - | - | - | - | - | - |
|  | - | 9 | 97951678 | - | - | - | - | - | - | - | - |
|  | - | 9 | 98248533 | - | - | - | - | - | - | - | - |
|  | - | 9 | 100892323 | - | - | - | - | - | - | - | - |
|  | - | 9 | 101096088 | - | - | - | - | - | - | - | - |
|  | - | 9 | 101404065 | - | - | - | - | - | - | - | - |
|  | - | 9 | 101924769 | - | - | - | - | - | - | - | - |
|  | - | 9 | 102174037 | - | - | - | - | - | - | - | - |
|  | - | 9 | 102216094 | - | - | - | - | - | - | - | - |
|  | - | 9 | 102423847 | - | - | - | - | - | - | - | - |
|  | - | 9 | 102561755 | - | - | - | - | - | - | - | - |
|  | - | 9 | 102562060 | - | - | - | - | - | - | - | - |
|  | - | 9 | 102636296 | - | - | - | - | - | - | - | - |
|  | - | 9 | 102937004 | - | - | - | - | - | - | - | - |
|  | - | 9 | 103166715 | - | - | - | - | - | - | - | - |
|  | - | 9 | 103457924 | - | - | - | - | - | - | - | - |
|  | - | 9 | 103469106 | - | - | - | - | - | - | - | - |
|  | - | 9 | 103479069 | - | - | - | - | - | - | - | - |
|  | - | 9 | 103682658 | - | - | - | - | - | - | - | - |
|  | - | 9 | 103776239 | - | - | - | - | - | - | - | - |
|  | - | 9 | 103832805 | - | - | - | - | - | - | - | - |
|  | - | 9 | 103884030 | - | - | - | - | - | - | - | - |
|  | - | 9 | 103929450 | - | - | - | - | - | - | - | - |
|  | - | 9 | 103938082 | - | - | - | - | - | - | - | - |
|  | rs144274933 | 9 | 103944315 | SMC2 | 150000 | intergenic | - | 0.18412 | - | - | - |
|  | rs186752055 | 9 | 103979368 | SMC2 | 115000 | intergenic | - | 0.18412 | - | - | - |
|  | - | 9 | 104080610 | - | - | - | - | - | - | - | - |
|  | - | 9 | 104189862 | - | - | - | - | - | - | - | - |
|  | - | 9 | 104219362 | - | - | - | - | - | - | - | - |
|  | - | 9 | 104223472 | - | - | - | - | - | - | - | - |
|  | rs199562704 | 9 | 104786394 | ABCA1 | 0 | intronic | 8.526 | 0.18412 | 4 | 4 | - |
|  | - | 9 | 105164953 | - | - | - | - | - | - | - | - |
|  | rs201343203 | 9 | 105477274 | FSD1L | 0 | intronic | - | 0.48029 | - | - | + |
|  | rs188280004 | 9 | 105670420 | TAL2 | 7333 | intergenic | 1.177 | 0.13454 | 5 | 15 | + |
|  | rs186868542 | 9 | 105725513 | TMEM38B | 0 | intronic | 1.876 | 0.5497 | 4 | 15 | + |
|  | - | 9 | 105756160 | - | - | - | - | - | - | - | - |
|  | - | 9 | 105801547 | - | - | - | - | - | - | - | - |
|  | - | 9 | 106070662 | - | - | - | - | - | - | - | - |
| Pomak - PDW | rs73183273 | 20 | 58478356 | APCDD1L | 0 | intronic | - | 0.88155 | - | - | - |
|  | - | 20 | 58654696 | - | - | - | - | - | - | - | - |
|  | - | 20 | 58657672 | - | - | - | - | - | - | - | - |
|  | - | 20 | 58790184 | - | - | - | - | - | - | - | - |
|  | rs190711845 | 20 | 58685286 | STX16-NPEPL1 | 0 | intronic | 7.562 | 1.0 | 4 | 5 | - |
|  | rs117112030 | 20 | 58796310 | PIEZO1P2 | 12487 | intergenic | 2.491 | 0.58955 | 5 | 14 | - |
|  | - | 20 | 58704921 | - | - | - | - | - | - | - | - |
| Manolis - RBC | - | 15 | 101913651 | - | - | - | - | - | - | - | - |
|  | - | 15 | 101904736 | - | - | - | - | - | - | - | - |

Chr: chromosome

Position: position on hg19

NearestGene: The nearest Gene of the SNP based on ANNOVAR annotations.

Distance: Distance to the nearest gene. SNPs which are locating in the gene body or 1kb up- or down-stream of TSS or TES have 0.

Function: Functional consequence of the SNP on the gene obtained from ANNOVAR.

CADD: CADD score which is computed based on 63 annotations.

RDB: RegulomeDB probability score (ranging from 0 to 1). 1 is the highest score that the SNP has the most biological evidence to be regulatory element.

minChrState: The minimum 15-core chromatin state across 127 tissue/cell type.

commonChrState: The most common 15-core chromatin state across 127 tissue/cell types.

Enhancer: Overlap with enhancer histone site, active in blood.

**Supplementary Table 3. Results of the credible sets in the latest GWAS of blood traits.**

|  | | | | | | **Astle et al. 2016 [PMID: 27863252]** | | | | **HELIC [this study]** | | | |
| --- | --- | --- | --- | --- | --- | --- | --- | --- | --- | --- | --- | --- | --- |
| **Trait** | **rsID** | **Chr** | **Position (b37)** | **REF** | **ALT** | **ALT_FREQ** | **Beta** | **SE** | **P** | **ALT_FREQ** | **Beta** | **SE** | **P** |
| **Pomak** | | | | | | | | | | | | | |
| white blood cell count | rs551751343* | 2 | 81711816 | C | T | 0.0005 | 0.05 | 0.13 | 0.688 | 0.004 | 1.72 | 0.29 | 4.14E-09 |
|  | **rs73941786** | 2 | 83062983 | A | C | 0.0046 | -0.07 | 0.03 | 0.017 | 0.004 | 1.72 | 0.29 | 4.14E-09 |
|  | rs190806297* | 2 | 83764938 | G | A | 0.0006 | -0.20 | 0.11 | 0.066 | 0.004 | 1.72 | 0.29 | 4.14E-09 |
|  | rs140340075* | 2 | 83889512 | A | C | 0.0003 | -0.01 | 0.16 | 0.929 | 0.004 | 1.72 | 0.29 | 4.14E-09 |
|  | rs188113595* | 2 | 84158113 | C | T | 0.0005 | -0.12 | 0.11 | 0.280 | 0.004 | 1.72 | 0.29 | 4.14E-09 |
|  | rs548781149* | 2 | 84386723 | C | T | 0.0003 | 0.23 | 0.18 | 0.203 | 0.004 | 1.72 | 0.29 | 4.14E-09 |
|  | rs531231069 | 2 | 82869328 | T | C | 0.0010 | -0.10 | 0.08 | 0.188 | 0.005 | 1.45 | 0.26 | 2.52E-08 |
| red cell distribution width | rs182113470 | 9 | 114643442 | A | G | 0.0015 | 0.09 | 0.06 | 0.167 | 0.002 | -2.64 | 0.39 | 3.92E-11 |
|  | rs138523839 | 9 | 114885019 | A | G | 0.0014 | -0.01 | 0.06 | 0.833 | 0.002 | -2.64 | 0.39 | 3.92E-11 |
|  | rs77635713 | 9 | 109362942 | C | T | 0.0121 | -0.01 | 0.02 | 0.680 | 0.003 | -2.47 | 0.37 | 1.01E-10 |
|  | rs74462705 | 9 | 109390054 | G | A | 0.0122 | 0.00 | 0.02 | 0.870 | 0.003 | -2.47 | 0.37 | 1.01E-10 |
|  | rs117587268 | 9 | 110095084 | C | G | 0.0011 | 0.02 | 0.06 | 0.792 | 0.003 | -2.26 | 0.35 | 2.30E-10 |
|  | rs773501657 | 9 | 111386116 | G | A | 0.0003 | -0.21 | 0.14 | 0.144 | 0.003 | -2.05 | 0.32 | 3.06E-10 |
|  | rs538815382 | 9 | 112182274 | G | A | 0.0002 | -0.27 | 0.15 | 0.067 | 0.003 | -2.05 | 0.32 | 3.06E-10 |
|  | rs141803223 | 9 | 112637054 | A | G | 0.0004 | -0.01 | 0.10 | 0.914 | 0.003 | -2.05 | 0.32 | 3.06E-10 |
|  | rs189173017* | 9 | 113215853 | A | G | 0.0045 | 0.01 | 0.03 | 0.588 | 0.004 | -1.89 | 0.30 | 8.42E-10 |
|  | rs369702397 | 9 | 114124394 | A | G | 0.0012 | 0.05 | 0.06 | 0.372 | 0.004 | -1.94 | 0.32 | 2.31E-09 |
|  | rs545561131 | 9 | 114146118 | C | T | 0.0012 | 0.06 | 0.06 | 0.342 | 0.004 | -1.94 | 0.32 | 2.31E-09 |
|  | rs562693440 | 9 | 113961915 | C | A | 0.0015 | 0.00 | 0.06 | 0.977 | 0.004 | -1.83 | 0.30 | 2.88E-09 |
|  | rs565079224 | 9 | 110012634 | G | A | 0.0130 | -0.03 | 0.02 | 0.127 | 0.004 | -1.76 | 0.29 | 3.20E-09 |
|  | rs569248438 | 9 | 100713960 | C | T | 0.0011 | 0.02 | 0.06 | 0.737 | 0.002 | -2.36 | 0.39 | 3.89E-09 |
|  | rs568091454 | 9 | 104687051 | G | A | 0.0019 | 0.03 | 0.05 | 0.565 | 0.002 | -2.36 | 0.39 | 3.89E-09 |
|  | rs530417185 | 9 | 104936319 | T | C | 0.0016 | 0.02 | 0.05 | 0.749 | 0.002 | -2.36 | 0.39 | 3.89E-09 |
|  | rs555777146 | 9 | 105398578 | T | C | 0.0021 | 0.04 | 0.05 | 0.424 | 0.002 | -2.36 | 0.39 | 3.89E-09 |
|  | rs559968830 | 9 | 105699286 | G | A | 0.0006 | 0.15 | 0.10 | 0.118 | 0.002 | -2.36 | 0.39 | 3.89E-09 |
|  | rs575509433 | 9 | 106444940 | T | C | 0.0005 | 0.00 | 0.09 | 0.957 | 0.002 | -2.36 | 0.39 | 3.89E-09 |
|  | rs562943353 | 9 | 106646311 | A | G | 0.0016 | 0.00 | 0.05 | 0.980 | 0.002 | -2.36 | 0.39 | 3.89E-09 |
|  | rs569604024 | 9 | 106691731 | A | G | 0.0006 | 0.04 | 0.09 | 0.605 | 0.002 | -2.36 | 0.39 | 3.89E-09 |
|  | rs144274933 | 9 | 106706596 | C | T | 0.0007 | 0.02 | 0.07 | 0.822 | 0.002 | -2.36 | 0.39 | 3.89E-09 |
|  | rs186752055 | 9 | 106741649 | A | G | 0.0008 | 0.02 | 0.06 | 0.753 | 0.002 | -2.36 | 0.39 | 3.89E-09 |
|  | rs574569400 | 9 | 106842891 | G | A | 0.0008 | 0.02 | 0.06 | 0.729 | 0.002 | -2.36 | 0.39 | 3.89E-09 |
|  | rs562332781 | 9 | 106952143 | G | A | 0.0008 | -0.01 | 0.06 | 0.895 | 0.002 | -2.36 | 0.39 | 3.89E-09 |
|  | rs540673651 | 9 | 106985753 | C | T | 0.0008 | 0.00 | 0.07 | 0.959 | 0.002 | -2.36 | 0.39 | 3.89E-09 |
|  | rs199562704 | 9 | 107548675 | C | T | 0.0003 | -0.13 | 0.15 | 0.384 | 0.002 | -2.36 | 0.39 | 3.89E-09 |
|  | **rs560612431** | 9 | 107927234 | A | T | 0.0022 | -0.10 | 0.04 | 0.026 | 0.002 | -2.36 | 0.39 | 3.89E-09 |
|  | rs188280004 | 9 | 108432701 | A | G | 0.0006 | -0.08 | 0.11 | 0.429 | 0.002 | -2.36 | 0.39 | 3.89E-09 |
|  | rs186868542 | 9 | 108487794 | G | T | 0.0025 | -0.02 | 0.04 | 0.542 | 0.002 | -2.36 | 0.39 | 3.89E-09 |
| platelet distribution width | rs73183273* | 20 | 57053412 | C | G | 0.0106 | -0.02 | 0.02 | 0.360 | 0.007 | 1.44 | 0.23 | 8.88E-10 |
|  | rs574441315 | 20 | 57232728 | G | C | 0.0025 | 0.04 | 0.04 | 0.408 | 0.009 | 1.24 | 0.21 | 4.06E-09 |
|  | rs190711845 | 20 | 57260342 | A | G | 0.0029 | 0.01 | 0.04 | 0.885 | 0.009 | 1.24 | 0.21 | 4.06E-09 |
|  | rs117112030 | 20 | 57371365 | A | T | 0.0034 | 0.02 | 0.03 | 0.594 | 0.009 | 1.20 | 0.20 | 5.24E-09 |

Chr: chromosome; Pos: position (b37); REF: reference allele; ALT: effect allele; ALT_FREQ: allele frequency of the effect allele; SE: standard error; P: p-value

The markers that have reached nominal significance threshold in previous GWAS are highlighted in bold. The star (*) denotes the lead variant in each region.

**Supplementary Table 5**. Case-only regression analysis comparing the effects of each individual *HBB* mutation on red cell traits to the most common thalassemia mutation: IVS-I-110 (c.93-21G>A) for MANOLIS and IVS-II-745 (c.316-106C>G) for Pomak. The analyses were adjusted for age and sex.

|  | **MANOLIS** |  |  |  | **Pomak** |  |  |  |
| --- | --- | --- | --- | --- | --- | --- | --- | --- |
| **Trait** | **Mutation** | **Beta** | **SE** | **P-value** | **Mutation** | **Beta** | **SE** | **P-value** |
| Red cell distribution width | HbS c.20A>T | 10.15 | 1.66 | 4.8E-8 | HbO-Arab c.364G>A | 5.58 | 0.89 | 4.2E-9 |
|  | IVS-I-6 c.92+6T>C | 9.14 | 2.55 | 6.1E-4 | IVS-I-6 c.92+6T>C | 4.02 | 1.38 | 4.1E-3 |
|  | CD39 c.118C>T | 0.29 | 1.47 | 0.84 |  |  |  |  |
|  | CD8/9+G c.27dupG | -0.81 | 1.32 | 0.54 |  |  |  |  |
| Red blood cell count | HbS c.20A>T | -0.84 | 0.18 | 8.4E-6 | HbO-Arab c.364G>A | -0.65 | 0.12 | 2.1E-7 |
|  | IVS-I-6 c.92+6T>C | -0.51 | 0.30 | 0.09 | IVS-I-6 c.92+6T>C | 0.05 | 0.19 | 0.79 |
|  | CD39 c.118C>T | 0.13 | 0.16 | 0.42 |  |  |  |  |
|  | CD8/9+G c.27dupG | -0.06 | 0.15 | 0.69 |  |  |  |  |
| Haemoglobin | HbS c.20A>T | 1.09 | 0.39 | 6.4E-3 | HbO-Arab c.364G>A | 2.59 | 0.38 | 3.2E-10 |
|  | IVS-I-6 c.92+6T>C | 0.35 | 0.65 | 0.59 | IVS-I-6 c.92+6T>C | 1.10 | 0.59 | 0.06 |
|  | CD39 c.118C>T | -0.09 | 0.35 | 0.79 |  |  |  |  |
|  | CD8/9+G c.27dupG | -0.57 | 0.33 | 0.09 |  |  |  |  |
| Haematocrit | HbS c.20A>T | 1.98 | 1.21 | 0.11 | HbO-Arab c.364G>A | 3.20 | 0.94 | 8.6E-4 |
|  | IVS-I-6 c.92+6T>C | 1.42 | 2.02 | 0.48 | IVS-I-6 c.92+6T>C | 3.16 | 1.46 | 0.03 |
|  | CD39 c.118C>T | 0.02 | 1.10 | 0.99 |  |  |  |  |
|  | CD8/9+G c.27dupG | -1.86 | 1.03 | 0.07 |  |  |  |  |
| Mean corpuscular volume | HbS c.20A>T | 14.92 | 1.39 | 6.2E-17 | HbO-Arab c.364G>A | 14.96 | 1.25 | 5.5E-23 |
|  | IVS-I-6 c.92+6T>C | 8.93 | 2.32 | 2.5E-4 | IVS-I-6 c.92+6T>C | 5.16 | 1.94 | 8.8E-3 |
|  | CD39 c.118C>T | -1.33 | 1.26 | 0.30 |  |  |  |  |
|  | CD8/9+G c.27dupG | -2.51 | 1.18 | 0.04 |  |  |  |  |
| Mean corpuscular haemoglobin | HbS c.20A>T | 5.88 | 0.47 | 1.9E-20 | HbO-Arab c.364G>A | 7.77 | 0.58 | 1.1E-26 |
|  | IVS-I-6 c.92+6T>C | 2.72 | 0.78 | 7.8E-4 | IVS-I-6 c.92+6T>C | 1.85 | 0.90 | 0.04 |
|  | CD39 c.118C>T | -0.53 | 0.42 | 0.21 |  |  |  |  |
|  | CD8/9+G c.27dupG | -0.78 | 0.39 | 0.05 |  |  |  |  |
| Mean corpuscular haemoglobin concentration | HbS c.20A>T | 1.11 | 0.34 | 1.8E-3 | HbO-Arab c.364G>A | 3.98 | 0.32 | 2.3E-24 |
|  | IVS-I-6 c.92+6T>C | -0.33 | 0.57 | 0.56 | IVS-I-6 c.92+6T>C | 0.47 | 0.49 | 0.34 |
|  | CD39 c.118C>T | -0.16 | 0.31 | 0.60 |  |  |  |  |
|  | CD8/9+G c.27dupG | 0.14 | 0.29 | 0.63 |  |  |  |  |

**Supplementary Table 6.** Pathogenic HBB mutations present in 3,724 individuals from INTERVAL and their associations with red cell distribution width

| **Mutation** | **Observed in HELIC** | **Position b38** | **Minor allele count** | **Beta** | **P-value** |
| --- | --- | --- | --- | --- | --- |
| rs63751128 c.*111A>G |  | 5225487 | 1 | 0.76 | 0.45 |
| rs33946267 (c.364G>A, p.Glu122Lys) | Pomak | 5225678 | 1 | -1.08 | 0.281 |
| rs33913413 (c.316-3C>A) | MANOLIS | 5225729 | 1 | -0.88 | 0.380 |
| rs11549407 (c.118C>T, p.Gln40Ter) | MANOLIS | 5226774 | 1 | -2.61 | 0.009 |

**Supplementary Figures**

**Supplementary Figure 1. Manhattan plot** showing genome-wide associations of variants with red cell distribution width in A) MANOLIS and B) Pomak. Genome-wide significant associations are highlighted in green and indicated by grey dotted lines.


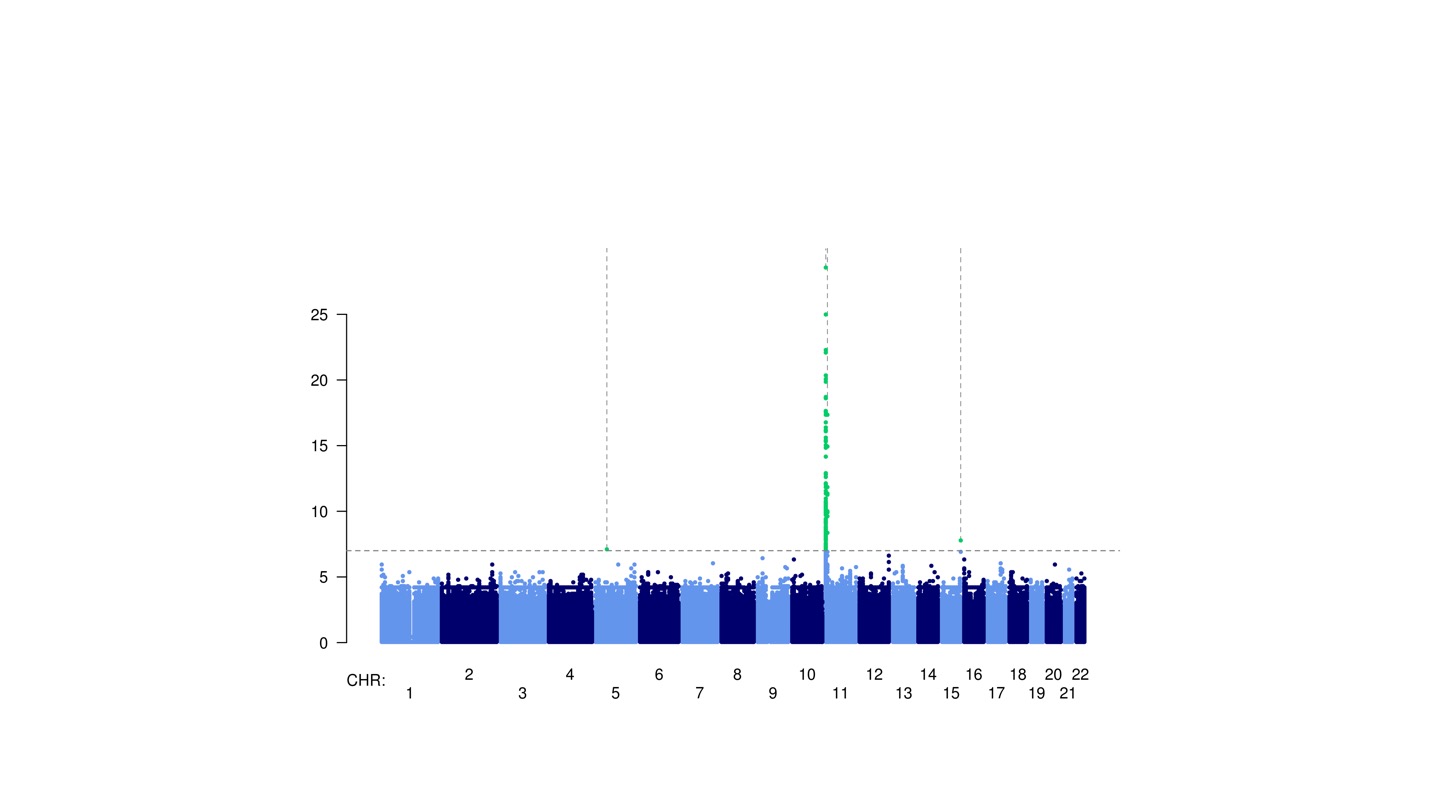


A)


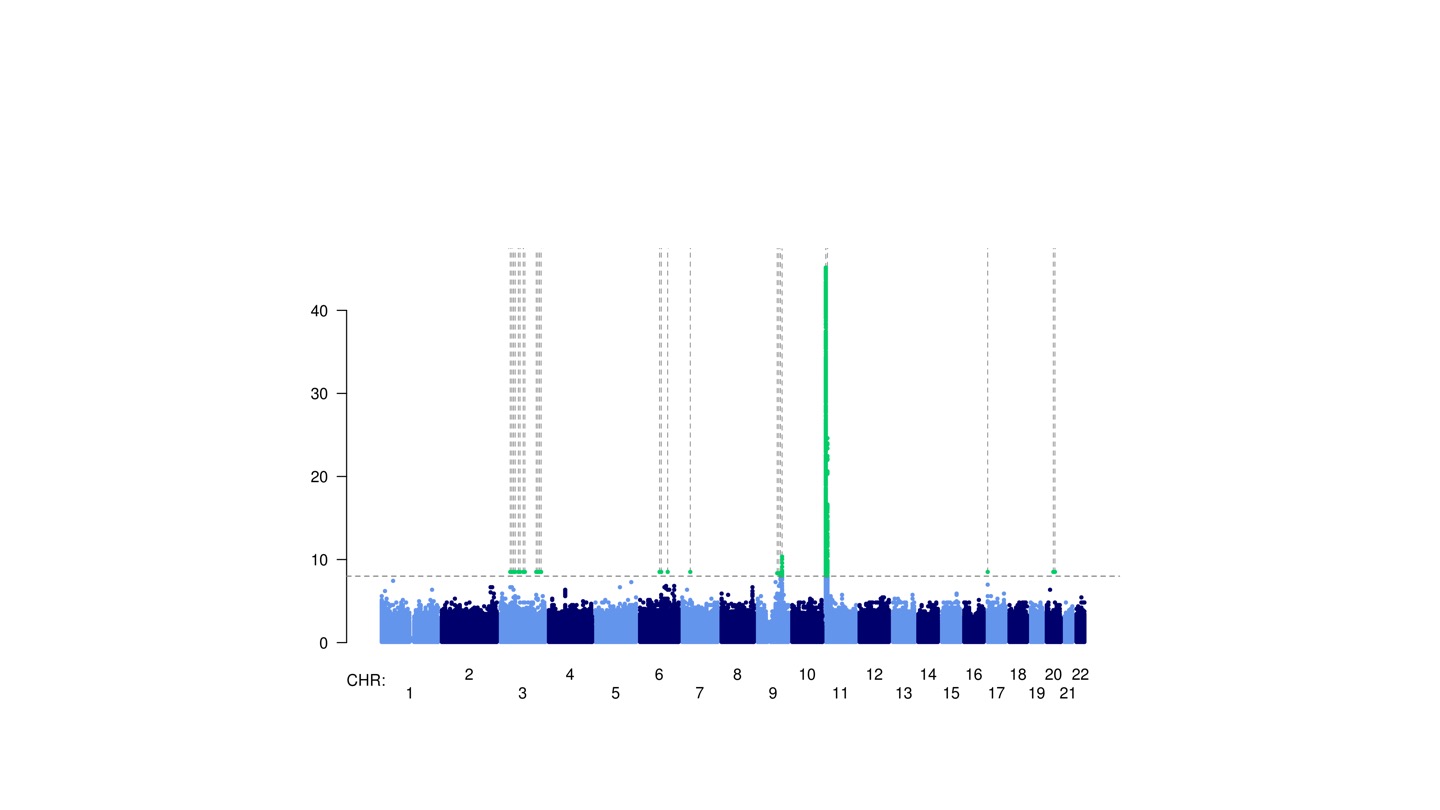


B)
